# Real-world treatment outcome of direct-acting antivirals and patient survival rates in chronic hepatitis C virus infection in Eritrea

**DOI:** 10.1038/s41598-023-47258-7

**Published:** 2023-11-27

**Authors:** Ghirmay Ghebrekidan Ghebremeskel, Michael Berhe Solomon, Oliver Okoth Achila, Samuel Tekle Mengistu, Rahel Frezghi Asmelash, Araia Berhane Mesfin, Mohammed Elfatih Hamida

**Affiliations:** 1 Nakfa Hospital, Northern Red Sea Ministry of Health Branch, Nakfa, Eritrea; 2 National Health Observatory Unit, Ministry of Health, Asmara, Eritrea; 3 Unit of Clinical Laboratory Science, Orotta College of Medicine and Health Sciences (OCMHS), Asmara, Eritrea; 4 Hamelmalo Community Hospital, Anseba Zone Ministry of Health Branch, Hamelmalo, Eritrea; 5 National Communicable Disease Control Division, Ministry of Health, Asmara, Eritrea; 6 Department of Medical Microbiology, Orotta College of Medicine and Health Sciences (OCMHS), Asmara, Eritrea

**Keywords:** Diseases, Health care, Medical research

## Abstract

Reliable real-world data on direct acting anti-retroviral (DAA) uptake and treatment outcomes are lacking for patients with hepatitis C virus (HCV) in sub-Saharan Africa. This study provides data on HCV DAA-based treatment outcomes, mortality, loss-to-follow up, and associated factors among patients in Eritrea. A multicenter retrospective observational cohort study was conducted in two tertiary hospitals in Asmara, Eritrea. A structured checklist was used to collect data from patient’s cards. Descriptive and inferential statistics used included means (± Standard deviation (SD), medians (Interquartile range (IQR), chi-squire (χ^2^), Kaplan–Meier estimates, and multivariate Cox proportional hazard models. A total of 238 patients with median age of 59 years (IQR 50–69 years) were enrolled in the study. Out of the 227 patients initiated on treatment, 125 patients had viral load measurements at 12 weeks after end of treatment (EOT) whereas 102 patients had no viral load measurements at 12 weeks EOT. Among the patients with HCV RNA data post-EOT 12, 116 (92.8%) had sustained viral response (SVR). The prevalence of death and loss-to-follow up (LTFU) were (7.5%, 95% CI 1.7–4.1) and 67 (28.1%, 95% CI 22.3–33.9) translating into an incidence of 1.1 (95% CI 0.8–1.5) per 10,000 person days. Independent predictors of LTFU included the enrollment year (2020: aHR = 2.2, 95% CI 1–4.7; *p* value = 0.04); Hospital (Hospital B: aHR = 2.2, 95% CI 1–4.7; *p* value = 0.03) and the FIB-4 score (FIB-Score < 1.45: aHR = 3.7, 95% CI 1.2–11.5; *p* value = 0.02). The SVR rates achieved in this cohort were high. However, high LTFU and high mortality driven largely by late presentation and suboptimal population screening/case finding, were uncovered. These challenges can be addressed by test-and-treat programs that simultaneously prioritize programmatic screening, decentralization of care, and better patient tracking in the HCV care cascade.

## Introduction

Hepatitis C virus (HCV), a predominantly blood-borne hepatotropic RNA virus with ~ 7 genotypes (Gt1-7) and > 67 confirmed subtypes^1^, has emerged as one of the leading causes of mortality and morbidity worldwide^2^. According to a recent World Health Organization (WHO) global estimate, the number of people with chronic HCV infection is approximately 71 million (95% confidence interval (CI): 62–79 million) people (1%)^3^ and only 20% are aware of their condition^4^. In terms of new infections, the data suggest that approximately 1.5 million new infections are registered per year (global incidence: 23.7 per 100,000)^5^. Most of these new infections have been attributed to iatrogenic causes, injection drug use, vertical transmission, body piercings/traditional scarification, and occupational exposure (e.g. needle-stick injuries), among others^6^.

In general, spontaneous clearance of the virus can occur within 6 months in approximately 30% (95% CI 15–45%) of infected persons^7^. However, 70% (95% CI 55–85%) progress to chronic HCV infection that can remain asymptomatic, and therefore unnoticeable, for decades^7^. Chronic hepatitis C (CHC) viremia is associated with multiple hepatic and extra hepatic sequelae which can lead to mortality^8–10^. Collectively, these complications were responsible for approximately 580,000 HCV-related deaths in 2017 and substantial impairments in multiple health-related quality of life (HRQL) indices^11,12^. Additional data suggests that unlike, Human Immunodeficiency virus (HIV), or tuberculosis (TB); mortality has trended upward in the last two decades. This upward trend is projected to increase in low- and middle-income countries (LMICs) if testing and subsequent treatments are not scaled-up^13^.

Fortunately, the development of multiple direct-acting antivirals (DAAs) that can achieve up to 95% cure rate with few adverse reactions has revolutionized treatment^2,14^. The main current therapeutic goal for HCV and prevention of liver disease progression is the sustained viral response (SVR)^15,16^. Beyond cure, SVR is associated with several solid clinical endpoints such as reduced likelihood of decompensated cirrhosis^12,17^. Additional public health benefits include better utility values and a reduction in community transmission rates^12^. In 2016, the potential benefit of effective screening programs and prompt treatment prompted the WHO to develop an improved public health action strategy to eliminate HCV infection by 2030^18^. This strategy calls for the diagnosis of 90% of infected individuals and the treatment of 80% of eligible patients.

Emerging evidence suggests that these goals are unlikely to be met^14^. First, some scholars have noted that the prevalence of HCV in LMIC in SSA is poorly researched^3,19^. Of note, the per-capita quantity and quality of real-world studies on DAA effectiveness or data on HCV care cascade performance are extremely rare. In Eritrea, real-world data on therapeutic outcomes is lacking. However, existing data point to low-to-moderate level of HCV infection^20,21^. These circumstances create a strong demand for updated high-quality data on a range of HCV-related issues in Eritrea. Therefore, this study was designed to investigate the treatment outcome of DAA therapy, estimate the frequency loss-to-follow up (LTFU), mortality and incidence rates, and associated factors in two pilot treatment centers in Asmara, Eritrea.

## Methods

### Study design and settings

**Figure 1:**
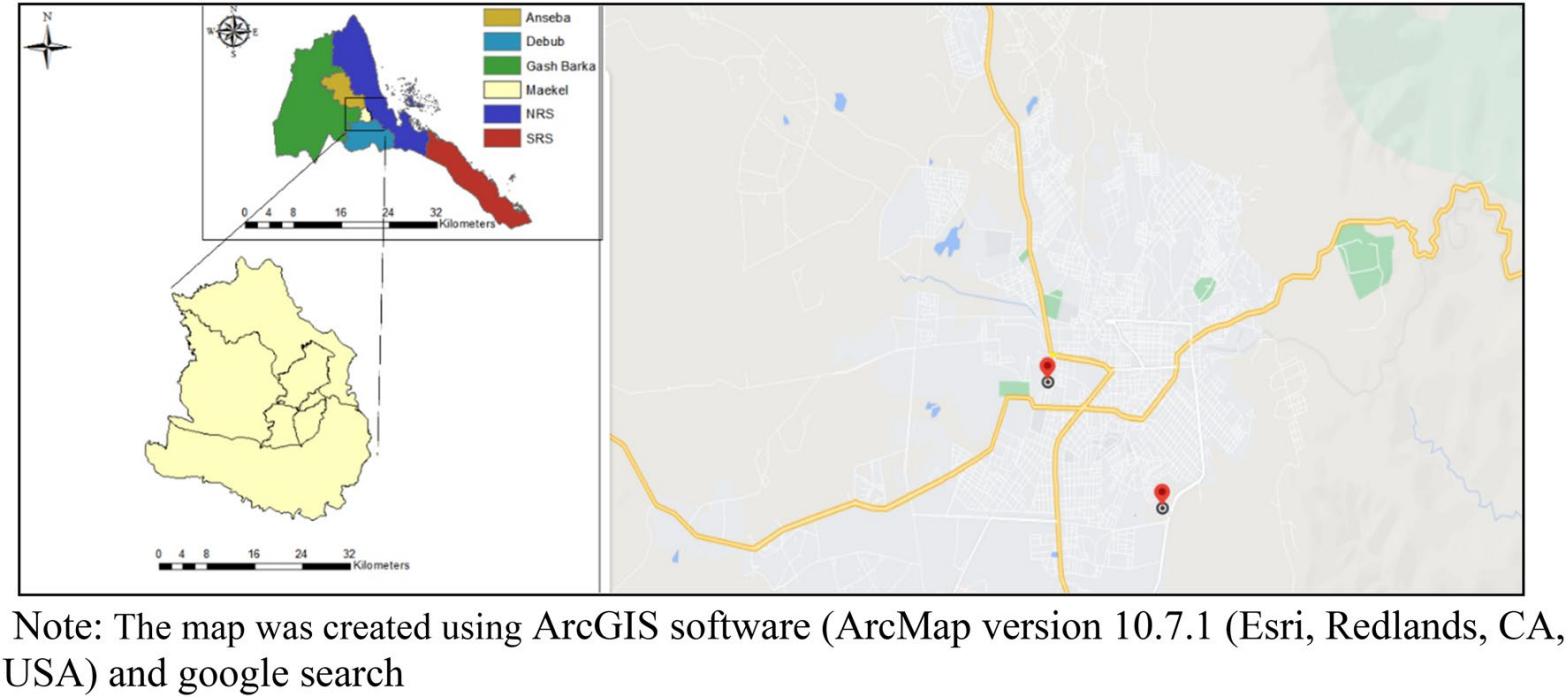
Map of Eritrea, Zoba Maekel (central zone) and locations of the treatment centers and HCV viral load testing center. *Note*: The map was created using ArcGIS software (ArcMap version 10.7.1 (Esri, Redlands, CA, USA) and google search [https://www.google.com/maps/place/Asmara,+Eritrea/@15.3329318,38.918554,16.25z/data=!4m6!3m5!1s0x166df23bb4c933a9:0xb8c1b327af63f5c5!8m2!3d15.3228767!4d38.9250517!16zL20vMGZuejg].

This observational retrospective cohort study was conducted on patients followed from 2018 to 2021 in the two major chronic HCV care centers in Asmara, Eritrea. See Fig. 1. At present, they serve as the major treatment centers for patients with HCV in Eritrea. Since the inception of the program in 2018, a total of 238 patients have received treatment and follow-up care in the two facilities. Treatment, in general, is guided by the Eritrean Ministry of Health (EMoH) Guideline for Chronic Hepatitis B and C infection (2018). Pan-genotypic DAA regimens recommended in the guideline include Sofosbuvir (SOF) (an NS5B Polymerase Inhibitor-nucleotide analogue), Daclatasvir (DCV) and Velpatasvir (VEL) (NS5A replication complex inhibitors) with SOF/VEL or SOF/DCV for 12 weeks being the preferred combination (See “Supplementary data” Page 2 for clinical laboratory assessments). The HCV viral load count was evaluated using HCV RNA assay. After initiation of DAA, HCV-RNA viral load assays are quantified at 12 and 24 weeks. Of note, these assessments are undertaken at the discretion of the attending physician/clinician and the costs of treatment are covered by the government.

### Participants

**Figure 2:**
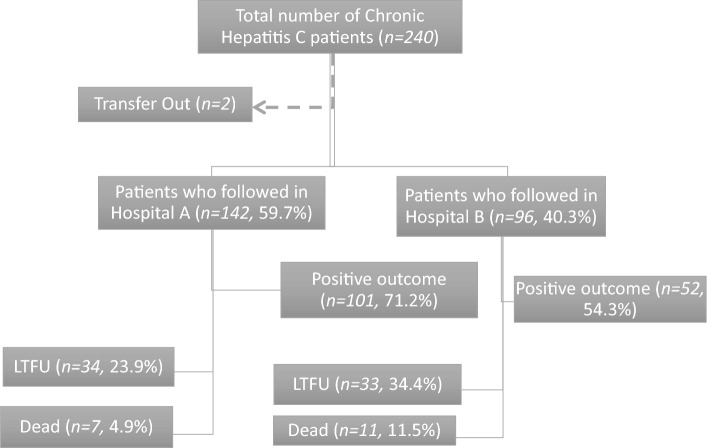
Flow diagram of study enrollment among the Chronic Viral Hepatitis C patients who followed in Tertiary hospitals in Asmara.

Patients in the two treatment centers are pooled from the entire country. In general, most patients are referred by clinicians in centers across the country or by transfusion centers following HCV antibody positivity on serological testing. In 2019, several patients were referred following screening campaigns among specific subgroups^22^. All patients aged > 18 years, registered in the two treatment centers were enrolled in this study. See Fig. 2 for additional details.

### Data collection tool

Data have been collected via a structured checklist from each patient’s clinical card that is routinely documented form for every patient upon enrollment and follow-up. The checklist was structured in a systematic way that would enable data collectors to retrieve data in an orderly fashion and detect systematic errors.

### Clinical and biochemical parameters

The following laboratory and clinical parameters were collected from the patient’s medical records: sex, age at enrollment, address, enrollment year, marital status, DAA regimen, HCV-RNA (baseline, 12 and 24 weeks), Platelets (PLTs), Alanine aminotransferase (ALT), Aspartate aminotransferase (AST), serum bilirubin (BIL), HIV status, Hepatitis B virus (HBV), and drug-side effect. Follow-up outcomes were also collected from clinical cards.

### Outcome measures and formulas

Patients were considered to have SVR if they had undetectable serum HCV RNA 12 weeks after EOT^16^. End of treatment (EOT) was estimated from the last day covered by prescription of SOF/DCV or SOF/VEL. Further, treatment nonresponse was defined as detectable HCV-RNA after EOT. Survival funtions included death and loss-to-follow up (LTFU). Death event was defined as all-cause mortality occurring during the patient’s follow up. In turn, loss-to-follow-up (LTFU) was defined as nonattendance of scheduled clinic appointments after enrollment into care^23^.

Cirrhosis probability was defined using FIB-4 (Non-invasive Fibrosis Evaluation in Hepatitis C) formula ([Age (years) × AST (U/L)]/[PLT (× 10^9^/L) × ALT^1/2^ (U/L)]. Computed scores were grouped as follows: Less likely (< 1.45 points), Indeterminate (1.45–3.25 points), and highly likely (> 3.25 points)^20^. In addition, the AST level with platelet ratio index (APRI) was also calculated: APRI = [(AST/upper limit of the normal AST level) × 100]/PLT (× 10^9^/L). Cirrhosis was defined as a cut-off > 0.5^24^.

### Geographical data and mapping

Zonal boundary data and latitude, and longitude coordinates were obtained from the National Statistical and Geography office in Eritrea. For coordinates that could not be obtained from this office, alternate sources ArcGIS software (ArcMap version 10.7.1 (Esri, Redlands, CA, USA)) and a Google search (See links in supplementary file 1) were employed.

### Data processing and analysis

Analysis was conducted using IBM SPSS (version 26) and STATA version 12.0 (STATA Corporation, College Station, TX). Descriptive statistics for categorical variables were analyzed using chi-square (χ^2^)/Fishers exact test and summarized using counts (frequency), proportions (percentages), means (± SD) and medians (interquartile range (IQR). Normality test (Kolmogorov–smirnov test) was conducted prior to any statistical computation and the appropriate parametric (t-test, ANOVA) and nonparametric statistics (Mann–Whitney U and Kruskal Wallis) were used to evaluate differences. Kaplan–Meier curves were used to estimate survival rates and failure rates at different intervals of follow-up. All LTFUs were censored on the date of their last visit. Multivariate Cox regression model was implemented for assessing the variables that predict LTFU. The final results are presented as adjusted hazard ratios (aHR) with a 95% CI. Two-sided *p* value < 0.05 was considered significant.

### Ethical consideration

Ethical approval was obtained from the Ministry of Health research ethics and protocol review committee with a letter of reference (Approval Number: Ref: 01/22). All the information gathered was de-identified, and at most confidentiality was upheld. As the study also included data based on patients’ clinical card records, consent for the data access was waived by the ethical committee in place of the patients. All procedures of the study also followed the recommendation of the Declaration of Helsinki Convention.

## Results

### Inter-facility analysis of cohort clinical and demographic characteristics

A total of 238 patients [Hospital A: 142(59.7%) vs. Hospital B: 96(40.3%)], treatment naïve patients, were enrolled for care from 2018 to 2021(94 (39.5%) ≤ 2019; 74(31.1%) = 2020; 70(29.4%) ≥ 2021). The median (IQR) age at diagnosis was 59 (IQR 50–69) years. HCV/HIV and HCV/HBV co-infections were observed in 9 (3.8%) and 3 (1.3%) patients, respectively. The mean (± SD) hemoglobin (Hgb) and PLTs count were 14.3 (± 1.7) g/dL and 181.9 (± 92.3) × 10^9^/µL, respectively. Moreover, anemia (Hgb < 12 g/dL in women and Hgb < 13 g/dL in men) and thrombocytopenia were present in 12 (5%) and 76 (31.9%) patients, respectively. According to FIB-4 score estimates, cirrhosis was highly likely in 81 (34%) patients. In contrast, 81 (34%) of the patients were in the indeterminate category whereas 47 (19.5%) of the patients had low likelihood of cirrhosis. See Table 1 for pairwise comparisons of means, medians and/or proportions in the two facilities.

**Table 1:** Demographic characteristics, patient history, and laboratory measurements at inclusion in the study among chronic Hepatitis-C infected individuals in Eritrea.

Characteristics	Population N (%)	Hospital A N (%)	Hospital B N (%)	*p* value (χ^2^)
Age at Enrollment (Mean ± SD)	59 ± 12.6	59 ± 12.7	60.5 ± 12.4	0.34^b^
< 60 years	109 (46)	71 (50.4)	38 (39.6)	0.1 (2.6)
≥ 60 years	128 (54.0)	70 (49.6)	58 (60.4)	
Gender				
Male	100 (42)	63 (44.4)	37 (38.5)	0.37 (0.798)
Female	138 (58)	79 (55.6)	59 (61.5)	
Address				
Central zone	199 (83.6)	119 (83.8)	23 (83.3)	0.92 (0.009)
Outside central zone	39 (16.3)	23 (16.2)	16 (16.7)	
Enrollment year				
≤ 2019	94 (39.5)	66 (46.4)	28 (29.2)	**0.026 (7.3)**
2020	74 (31.1)	38 (26.8)	36 (37.5)	
≥ 2021	70 (29.4)	38 (26.8)	32 (33.3)	
Marital status				
Married	98 (83)	17 (60.7)	81 (91)	< **0.001 (21.5)**
Single	19 (17.1)	11 (39.3)	11 (8.2)	
DAA regimen				
SOF/VEL	183 (76.9)	90 (63.4)	93 (96.9)	< **0.001 (36.3)**
SOF/DCV	44 (18.5)	41 (28.9)	3 (3.1)	
Not initiated on DAA	11 (4.6)	11 (7.7)	0	
Median Baseline HCV RNA [IQR]) (Log10 IU/ml)	6.12 (5.5–6.5)	6.1 (5.7–6.6)	6 (5.4–6.5)	0.15^a^
HCV RNA (IU/ml)				
< 800,000	80(38.5)	49(61.2)	31(38.8)	0.37(0.982)
> 800,000	128(61.5)	87(68.0)	41(32.0)	
HBsAg				
Positive	3 (1.3)	2 (1.4)	1 (1)	< **0.001 (27.9)**
Negative	73 (30.7)	62 (43.7)	11 (11.5)	
No data entry	162 (68.1)	78 (54.9)	84 (87.5)	
HIV status				
Positive	9 (3.8)	5 (3.6)	4 (4.2)	0.58 (0.29)
Negative	84 (35.8)	48 (34.5)	36 (37.5)	
No data entry	142 (60.4)	86 (61.9)	56 (58.3)	
Baseline Hematology				
Hemoglobin (g/dL), mean ± SD	14.3 ± 1.7	14.3 ± 1.6	- (Only one)	
Platelets (× 10^9^/µL), mean ± SD	181.9 ± 92.3	179.3 ± 68.9	190.5 ± 81.5	0.6^b^
Liver function test				
AST (IU/L) [median (IQR)]	41 (29–75)	45 (30–68)	40 (29–65.8)	0.65^a^
ALT (IU/L) [median (IQR)]	33.5 (19.8–60.2)	31 (17–60)	37.5 (22–64.5)	0.1^a^
(FIB-4) Non-invasive Fibrosis Evaluation in Hepatitis C				
Less likely	47 (19.5)	29 (26.4)	20 (23.5)	0.88 (0.246)
Intermediate	81 (34)	40 (36.4)	31 (36.5)	
Highly likely	81 (34)	41 (37.3)	34 (40)	
APRI score	0.5 (0.3–1.1)	0.5 (0.3–1.1)	0.5 (0.3–0.9)	0.5^a^
< 0.5	118 (55.1)	68 (54.4)	50 (56.2)	0.7 (0.06)
> 0.5	96 (44.9)	57 (45.6)	39 (43.8	
Treatment response				
Sustained virologic response	116 (92.8)	80 (95.2)	36 (87.8)	0.1 (1.3)
Treatment non-response	9 (7.5)	4 (4.8)	5 (12.2)	
Median Duration of treatment (IQR) weeks	90 (60–114)	89 (60–97)	94 (66–177)	**0.04**^a^
Survival outcome				
Positive outcome	153 (64.2)	101 (71.1)	52 (54.2)	**0.01 (8)**
Loss to follow up	67 (28.1)	34 (23.9)	33 (34.4)	
Dead	18 (7.5)	7 (4.9)	11 (11.5)	
Median duration of follow up, (IQR)	107 (60–239)	115 (89–275)	112 (69–181)	**0.01**^a^

SOF/DCV, Sofosbuvir/Daclatasvir; SOF/VEL, Sofosbuvir/Velpatasvir; HBsAg, Hepatitis B surface antigen; AST, Aspartate aminotransferase; ALT, Alanine aminotransferase; IQR, Interquartile range; SD, Standard deviation; DAA, Direct-acting antiretroviral therapy; HCV, Hepatitis C virus; RNA, Ribonucleic acid; HIV, Human Immunodeficiency virus.

^a^Mann-Whitney U test; ^b^independent samples t-test.

Significant values are in [bold].

### Specific host factors and liver Fib-4 score stages

Compared to participants with FIB-4 > 3.25 points, participants with FIB-4 < 3.25 points were younger (50 ± 12 vs. 60 ± 10 years, *p* value  < 0.001); had lower median AST (IQR) (27(20–34) vs. 66 (45–133) IU/L, *p* value  < 0.001); higher baseline Hgb (14.7 ± 1.7 g/dL) vs. 13.6 ± 1.6 g/dL) *p* value  < 0.02; and higher mean PLT counts (255(± 99) × 10^9^/µL) vs. 135(± 48) × 10^9^/µL), *p* value  < 0.001. Moreover, majority of patients with cirrhosis (FIB-4 score > 3.25) were initiated on SOF/VEL (77.9%). (See additional information in Table 2).

**Table 2:** Host factors and Fib-4 Score stages among chronic hepatitis-C infected individuals in Eritrea.

Characteristics	Cirrhosis less likely	Indeterminate	Cirrhosis highly likely	*P* value
Male n (%)	21(23.3)	34(37.8)	35(38.9)	0.956
Age in years, mean (± SD)	50 (12)	60 (10)	64 (10)	< 0.001^a^
Baseline AST (IU/L), median (IQR)	27 (20–34)	38 (30–52)	66 (45–133)	< 0.001^b^
Baseline ALT (IU/L), median (IQR)	26 (17–47)	38 (20–60)	33 (21–76)	0.1^b^
PLT (× 10^9^/µL) count, mean (± SD)	255 (99)	198 (56)	135 (48)	< 0.001^a^
Baseline Log_10_VL (IU/ML), median (IQR)	6.06 (5.3–6.6)	6.2 (5.7–6.6)	6 (5.6–6.3)	0.2^b^
HGB (g/dL), mean (± SD)	14.7 (1.7)	14.8 (1.1)	13.6 (1.6)	0.02^a^
DAA, SOF/VEL	38 (80.9%)	68 (85%)	60 (77.9%)	0.5^c^
DAA, SOF/DCV	9 (19.9%)	12 (15%)	17 (22.1%)	

AST, Aspartate aminotransferase; ALT, Alanine aminotransferase; IQR, interquartile range; HGB, hemoglobin; DAA, Direct acting antiviral therapy; Sofosbuvir/Daclatasvir; SOF/VEL, Sofosbuvir/Velpatasvir.

^a^One-way ANOVA, ^b^Kruskal-Wallis test.

### Treatment outcomes of DAA Therapy

**Figure 3:**
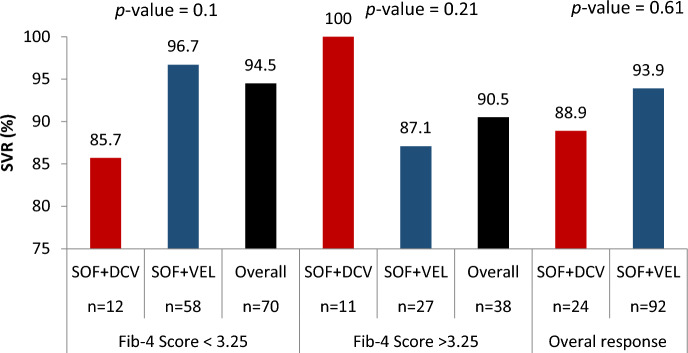
SVR attainment by specific DAA combinations per specific Fib-4 categories. a—Chi-square test.

Out of 238 patients enrolled in the study, 227 were initiated on treatment. The median (IQR) duration of treatment was 90 (IQR: 60–114) days. Of 227 patients who were placed on treatment with DAA, 125 (55%) had viral load measurements at 12 weeks EOT whereas 102 patients had no viral load measurements at 12 weeks EOT. In addition, 54 were LTFU and 18 died before SVR12 while 19 patients were on treatment during the study period. Of the 125 patients with HCV RNA data post EOT, 116 (92.8%) had SVR12. According to the data, there was no statistical difference in SVR rates between the SOF/VEL and SOF/DCV groups (93.9% and 88.9% respectively, *p* value  = 0.6). Moreover, 58 (96.7%) and 12 (85.7%) of patients on SOF/VEL and SOF/DOC with FIB-4 score < 3.25 attained SVR, respectively. Among patients with a FIB-4 score > 3.25 (Cirrhosis), 27 (87.1%) of patients on SOF/VEL and 11 (100%) of patients on SOF/DOC attained SVR. No significant difference was identified in the rate of SVR between the FIB-4 ≤ 3.25 and FIB-4 > 3.25 groups (94.5% and 90.5%, respectively, *p* value  = 0.4). (See Fig. 3 for details).

### Pre- and Post-treatment values of specific variables

Pre-treatment and post-treatment analysis of specific laboratory variables demonstrated that AST and ALT were significantly lower than pre-treatment values (41(IQR: 30–68) vs. 30(IQR: 24–35), *p* value  < 0.001) and (33(IQR: 20–60) vs. 18(IQR: 14–26), *p* value  < 0.001), respectively. Moreover, FIB-4 score and APRI score were significantly lower following treatment with DAA (2.1(IQR: 1.4–4.1) vs. 1.9 (IQR: 1.2–2.9), *p* value  = 0.003) and (0.5(IQR: 0.3–1) vs. 0.3 (IQR: 0.2–0.5), *p* value  < 0.001, respectively. However, PLT count demonstrated limited improvement following treatment (See Table 3 for details).

**Table 3:** Alterations in laboratory parameters in response to DAA among chronic hepatitis-C infected individuals in Eritrea.

Characteristics	Pretreatment	Post-treatment	*p* value
AST, median (IQR)	41 (30–68)	30 (24–35)	< **0.001**^a^
ALT, median (IQR)	33 (20–60)	18 (14–26)	< **0.001**^a^
PLT count, mean (± SD)	185 (78)	180 (69)	0.4^b^
VL Log_10_, median (IQR)	6.15 (5.56–6.58)	0.0 (0.0–0.0)	< **0.001**^a^
FiB-4 score	2.1 (1.4–4.1)	1.9 (1.2–2.9)	**0.003**^a^
APRI score	0.5 (0.3–1)	0.3 (0.2–0.5)	< **0.001**^a^

AST, Aspartate aminotransferase; ALT, Alanine aminotransferase. IQR, interquartile range; VL, Viral load; SD, Standard deviation.

^a^Wilcoxon signed-rank test, ^b^Paired Samples t-test.

Significant values are in [bold].

### Loss to follow-up and Mortality in the HCV care cascade

**Figure 4:**
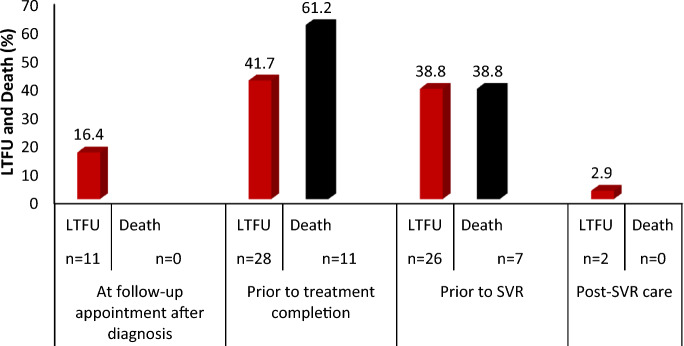
Proportion of loss to follow-up and Mortality in the Consensus HCV care cascade.

Death occurred in 18 (7.5% (95% CI 1.7–4.1) patients while 67 cases were LTFU (28.1%, 95% CI 22.3–33.9). Analysis of the proportion of LTFU and death through HCV Cascade of Care demonstrated that majority of LTFU and mortality occurred prior to treatment completion—28 (41.7%) and 11 (61.2%)), respectively. Furthermore, 26 (38.8%) were LTFU − 11 (16.4%) LFTU occurred at a follow-up appointment after diagnosis; 28 (41.7%) occurred before treatment completion; 26 (38.8%) occurred before SVR and 2 (2.9%) occurred post SVR care (Fig. 4).

### Factors associated with mortality and LTFU

Table 4 presents factors associated with mortality and LTFU. In this analysis, patients who died had a significantly shorter median duration on treatment with DAA of 31 days (IQR 21–227). In contrast, patients with positive outcomes and LFTU had longer duration of treatment—90 days (IQR 80–144) vs. 84 days (IQR 42–92), respectively (*p* value = 0.002). Moreover, patients who died had a higher median FIB-4 score, 6.9 (IQR 4.5–6.9) as compared to positive outcome 3.1 (IQR 1.6–6.2) and LTFU 1.3 (IQR: 0.9–6); *p* value  < 0.001. Similarly, mortality was associated with significantly higher median APRI and baseline AST. In contrast, LTFU was higher in both patients who had not started treatment and had been on follow-up for ≤ 12 weeks while Fib-score was relatively lower.

**Table 4:** Demographic characteristics, patient history, and laboratory measurements with treatment and follow-up outcome at inclusion time in the study among Hepatitis-C infected individuals in Eritrea.

Characteristics	Positive outcome n (%)	LTFU (%)	Dead (%)	*p* value
Gender				
Male	61 (39.9)	30 (44.8)	9 (50)	0.61 (0.9)
Female	92 (60.1)	37 (55.2)	9 (50)	
Age at enrollment, mean ± SD	58 (10)	50 (16)	58 (6)	0.34^a^
Address				
Central Zone	131 (85.6)	55 (82.1)	13 (72.2)	0.3 (2.2)
Outside central zone	22 (14.4)	12 (17.9)	5 (27.8)	
Enrollment year, median (IQR)	2019 (2019–2020)	2019 (2019–2019)	2018 (2018–2019	0.94^b^
< 2020	62 (40.5)	25 (37.3)	7 (38.9)	0.76 (1.8)
2020	44 (28.8)	25 (37.3)	5 (27.8)	
> 2020	47 (30.7)	17 (25.4)	6 (33.3)	
Marital status				
Married	55 (77.5)	31 (93.9)	12 (92.3)	0.07 (5.2)
Single	16 (22.5)	2 (6.1)	1 (7.7)	
Occupation				
Employed	41 (58.6)	13 (43.3)	8 (66.7)	0.2 (2.6)
Unemployed	29 (41.4)	17 (56.7)	4 (33.3)	
DAA regimen				
SOF/VEL	118 (77.6)	50 (73.5)	14 (82.4)	< **0.001 (25)**
SOF/DCV	34 (23.4)	7 (10.2)	3 (17.6)	
Not started on DAA	0 (0)	11 (16)	0	
Treatment duration, median (IQR)	90 (80–144)	84 (42–92)	31 (26–227)	**0.002**^b^
≤ 12 weeks	110 (72.4)	39 (90.7)	9 (75)	**0.003 (15.8)**
13–24 weeks	32 (21.1)	1 (2.3)	0	
> 24 weeks	10 (6.6)	3 (7)	3 (25)	
HBsAg status				
Positive	0	1 (1.5)	2 (11.1)	< **0.001 (18.8)**
Negative	53 (34.6)	17 (25.4)	3 (16.7)	
No data entry	100 (65.4)	49 (73.1)	13 (72.2)	
HIV status				
Positive	6 (4)	2 (3)	1 (5.6)	0.8 (0.9)
Negative	52 (34.4)	27 (40.9)	5 (27.8)	
No data entry	93 (61.6)	37 (56.1)	12 (66.7)	
FIB-4 score	3.1 (1.6–6.2)	1.3 (0.9–6)	6.9 (4.5–6.9)	**0.001**^b^
Less likely	30 (20.8)	16 (32.7)	1 (6.3)	**0.01 (13)**
Indeterminate	62 (43.1)	16 (32.7)	3 (18.8)	
Highly likely	52 (36.1)	17 (34.7)	12 (75)	
APRI score	0.5 (0.3–1)	0.4 (0.2–0.8)	1.1 (0.8–2.5)	< **0.001**^b^
< 0.5	84 (57.9)	32 (62.7)	2 (11.1)	< **0.001 (15.7)**
> 0.5	61 (42.1)	19 (37.3)	16 (88.9)	
Initial Serum HCV/RNA, Log_10_VL (IU/ML), median (IQR)	6.2 (5.6–6.5)	6 (5.4–6.5)	5.8 (4.9–6.2)	0.28^b^
Baseline hematology				
Hemoglobin (g/DL), mean ± SD	14.4 (1.6)	14.3 (1.7)	12.3 (1.6)	0.14^a^
Platelet count × 10^9^/µL, mean ± SD	181 (98)	188 (58)	133 (44)	0.49^a^
Baseline ALT (IU/L), median (IQR)	27.5 (19.7–57.7)	23 (13.2–39.5)	43.5 (35–43.5)	0.1
ALT < 40	82 (53.7)	33 (58.9)	6 (40)	0.39 (1.8)
ALT > 40	61 (42.7)	23 (41.1)	9 (60)	
Baseline AST (IU/L), median (IQR)	48.5 (31.7–73.2)	28 (21.7–65.7)	111.5 (53–111.5)	< **0.001**^b^
AST < 40	77 (52)	30 (55.6)	0	< **0.001 (17.8)**
AST > 40	71 (48)	24 (44.4)	17 (100)	
Duration of follow-up, median (IQR)	132 (91–264)	60 (2–121)	49 (14–228)	< **0.001**^b^

SD, Standard Deviation; IQR, interquartile range; HBsAg, Hepatitis B surface antigen; HIV, Human immunodeficiency virus; HCV, Hepatitis C virus; ALT, alanine aminotransferase; ASP, Aspartate Aminotransferase; SOF/DCV, Sofosbuvir/Daclatasvir; SOF/VEL, Sofosbuvir/Velpatasvir.

^a^One-way ANOVA, ^b^Kruskal-Wallis test.

Significant values are in [bold].

### Incidence rates, rate ratio and Kaplan–Meier survival estimates for mortality

**Figure 5:**
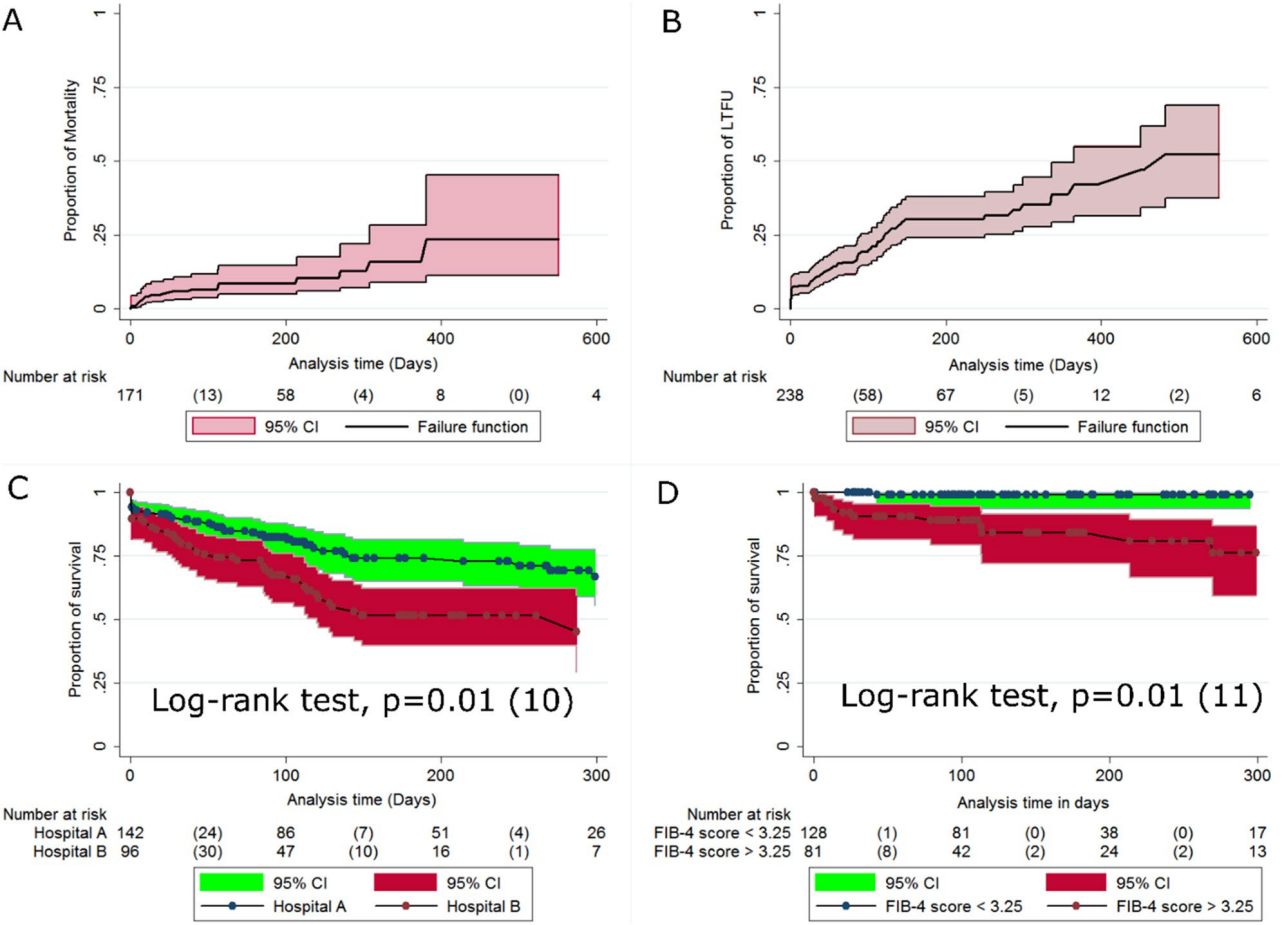
Kaplan–Meier curves for cumulative survival, LTFU and mortality of chronic HCV patients followed in the two major treatment centers in Eritrea from 2018-to 2021. (**A**) Overall cumulative proportion of death; (**B**) Overall cumulative proportion of LTFU; (**C**) Cumulative proportion of survival by hospital (**D**) Cumulative mortality curve by FIB 4 score.

Following 29,786 person-days of follow-up,incidence rate of death was 6.04 (95% CI 3.8–9.5) per 10,000 person-days. Kaplan–Meier survival analysis demonstrated that patients enrolled in Hospital B had a significantly shorter mean duration of survival (548 days (95% CI 403–694) vs. 578 days (95% CI 533–624), *p* value  = 0.02) (Fig. 5c) and higher risk of death, 3.3(95% CI 1.1–10.1). Moreover, patients with FIB-4 ≥ 3.25 score had a significantly shorter mean survival duration as compared to FIB-4 < 3.25 (472 days (95% CI 402–542) vs. (649 (95% CI 537–761) days), *p* < 0.001) (Fig. 5d). Lastly, patients from outside central zone had a shorter mean duration of survival, 358 days (95% CI 283–433) vs. 623 days (95% CI 547–669) for those from central zone, *p* < 0.05) (See Table 5 for details). Overall survival curve for mortality is displayed in Fig. 5a.

**Table 5:** Incidence rate and rate ratios of mortality, and Kaplan–Meier survival estimates among chronic Hepatitis-C patients in Eritrea.

Cohort Characteristics	Person time (days)	death Events	Incidence of death per 10,000 PDs (95% CI)	Relative risk rates (95% CI)	Mean survival duration in days (95% CI)	*p* value (log-rank)
Population	29,786	18	6.04 (3.8–9.5)			–
Hospital						
Hospital A	20,253	7	3.4 (1.6–7.20	1	578 (533–624)	**0.02 (5.4)**
Hospital B	9533	11	11.5 (6.3–20.8)	3.3 (1.1–10.1)	548 (403–694)	
Gender						
Female	15,691	9	5.7 (2.9–11)	1	526 (471–581)	0.91 (0.01)
Male	14,095	9	6.3 (3.3–12.2)	1.01 (0.3–2.8)	585 (459–712)	
Address						
Central zone	25,577	13	5 (2.9–8.7)	1	623 (547–669)	**0.05 (3.6)**
Outside central zone	4209	5	11.8 (4.9 -28.5)	2.3 (0.6–7)	358 (283–433)	
Enrollment year						
≤ 2019	14,824	7	4.7 (2.2–9.9)	1	624 (563–720)	0.17 (3.53)
2020	9469	5	5.2 (2.1–12.6)	1.2 (0.3–4.1)	468 (392–544)	
≥ 2021	5493	6	10.9 (4.9–24.3)	2.1 (0.6–6.70	477 (365–588)	
Initial regimen						
SOF/DCV	22,969	15	6.5 (3.9–10.8)	1	134 (30–238)	0.157 (3.7)
SOF/VEL	6816	3	4.4 (1.4–13.6)	0.67 (0.12–2.4)	191 (113–270)	
Treatment duration						
≤ 12 weeks	17,070	15	6.8 (4.1–11.30	1	79 (22–135)	0.1 (3.9)
13 and ≤ 24 weeks	7597	0	0	0	0	
> 24 weeks	4341	3	5.6 (1.8–17.4)	1 (0.2–3.80	319 (179–459)	
AST						
≤ 40 IU/L	16,406		0 (0)		0	< **0.001 (13.7)**
> 40 IU/L	17,612	17	7.4 (4.4–12)		144 (58–230)	
ALT						
≤ 40 IU/L	18,769	6	2.5 (1.1–6)	1	499 (457–542)	0.35 (0.86)
> 40 IU/L	15,154	9	4.6 (2.3–9.1)	1.8 (0.5–6.3)	572 (444–699)	
Non-invasive Fibrosis Evaluation in Hepatitis C (FIB-4)						
Cirrhosis is not highly likely	20,879	4	1.9 (0.7–5.1)	1	649 (537–761)	< **0.001 (11.3)**
Cirrhosis highly likely	12,176	12	9.8 (5.5–17.30	5.1 (1.5–21.8)	472 (402–542)	

ALT, alanine aminotransferase; AST, Aspartate Aminotransferase; SOF/DCV, Sofosbuvir/Daclatasvir; SOF/VEL, Sofosbuvir/Velpatasvir.

Significant values are in [bold].

### Incidence rates and Kaplan–Meier survival estimates for LTFU

The number of LTFU events was 67 events translating into an incidence rate of 1.1(95% CI 0.8–1.5) per 10,000 person-days. In the Kaplan–Meier analysis, patients enrolled at Hospital B had a shorter mean duration of survival, 304 days (95% CI 215–392) vs. 736 days (95%:515–956) in Hospital A, *p* < 0.001. Moreover, patients not initiated on DAA had significantly shorter mean survival duration than those on treatment, 169 days (95% CI 1–458) vs. 371 days (95% CI 308–434) for patients on SOF/DCV and 830 days (95% CI 659–1001) for patients on SOF/VEL, *p* < 0.001 (Table 6). Overall survival curve for LTFU is displayed in Fig. 5b.

**Table 6:** Incidence rate and Kaplan–Meier survival estimates for LTFU among chronic Hepatitis-C patients in Eritrea.

Cohort Characteristics	Person time (Days)	Events	Incidence of LTFU per 1000 PDs (95% CI)	Mean survival duration in days, 95% CI	*p* value (log-rank)
Population	35,490	67	1.1 (0.82–1.5)		–
Hospital					
Hospital A	24,719	34	0.78 (0.52–1.18)	736 (515–956)	**0.008 (7)**
Hospital B	10,771	33	1.8 (1.2–2.7)	304 (215–392)	
Gender					
Male	17,432	30	0.93 (0.6–1.45)	685 (448–921)	0.5 (0.45)
Female	18,058	37	1.3 (0.85–1.86)	376 (312–439)	
Address					
Central zone	30,604	55	1.04 (0.75–1.44)	700 (510–890)	0.45 (0.56)
Outside central zone	4886	12	1.3 (0.68–2.7)	267 (196–338)	
Enrollment year					
≤ 2019	18,689	25	0.69 (0.42–1.13)	695 (460–929)	0.18 (3.4)
2020	11,036	25	1.5 (0.95–2.4)	325 (248–401)	
≥ 2021	5765	17	1.8 (1–3.2)	373 (264–482)	
Initial regimen					
SOF/DCV	26,076	50	1.9 (1.4–2.5)	371 (308–434)	< **0.001 (27.7)**
SOF/VEL	7862	7	0.8 (0.4–1.8)	830 (659–1001)	
Not started	1552	10	6.4 (3.4–11.9)	169 (1–458)	
Treatment duration					
≤ 12 weeks	20,454	39	1.8 (1.3–2.5)	364 (290–438)	< **0.001 (16.1)**
13 and ≤ 24 weeks	7884	1	1.2 (0.1–9)	563 (479–646)	
> 24 weeks	4382	3	5.6 (1.8–17.4)	457 (338–575)	
AST					
≤ 40 mg/dL	16,406	30	1.3 (0.84–1.95)	404 (342–466)	0.73 (0.1)
> 40 mg/dL	15,242	24	0.7 (0.45–1.2)	389 (301–477)	
ALT					
≤ 40 mg/dL	18,335	33	1.3 (0.89–1.9)	565 (404–725)	0.26 (1.25)
> 40 mg/dL	13,291	23	0.68 (0.39–1.2)	407 (316–498)	
Non-invasive Fibrosis Evaluation in Hepatitis C (FIB-4)					
Less likely	6232	16	1.6 (0.94–2.9)	270 (169–371)	0.22 (3)
Intermediate	13,183	16	0.6 (0.33–1.24)	447 (374–521)	
Highly likely	11,300	17	0.81 (0.44–1.5)	396 (309–483)	

ALT, alanine aminotransferase; AST, Aspartate Aminotransferase; SOF/DCV, Sofosbuvir/Daclatasvir; SOF/VEL, Sofosbuvir/Velpatasvir.

Significant values are in [bold].

### Independent predictors of LTFU in chronic hepatitis C patients in Eritrea

Table 7 presents unadjusted and adjusted hazard ratios for variables associated with LTFU in 238 chronic HCV patients followed from 2018 to 2021. In the adjusted model, independent predictors of LTFU included enrollment year (2020: aHR = 2.2, 95% CI 1–4.7; *p* value = 0.04); Hospital (Hospital B: aHR = 2.2, 95% CI 1–4.7; *p* value = 0.03) and FIB-4 score (≥ 3.25: aHR = 3.7, 95% CI 1.2–11.5; *p* value  = 0.02).

**Table 7:** Cox proportional hazards of LTFU among chronic hepatitis C patients followed in ONRH and HNRH (2018–2021).

Characteristics	Unadjusted HR (95% CI)	*p* value	Adjusted HR (95% CI)	*p* value
Gender				
Male	*1 (Ref)*	0.7		
Female	1.1 (0.5–2.5)			
Age at enrollment	1 (0.9–1.05)	0.7		
Address				
Maekel	*1 (Ref)*	0.3		
Outside Maekel	1.3 (0.7–2.5)			
Hospital				
Hospital A	*1 (Ref)*		*1 (Ref)*	
Hospital B	1.9 (1.1–3.1)	**0.01**	2.2 (1–4.7)	**0.03**
Enrollment year				
< 2020	*1 (Ref)*		*1 (Ref)*	
2020	1.6 (0.9–2.8)	0.09	2.1 (1–4.4)	**0.04**
> 2020	1.7 (0.9–3.3)	0.08	2.5 (0.8–7.9)	0.1
HBsAg testing				
Known	*1 (Ref)*	0.8		
Not data entry	1 (0.4–2.6)			
HIV status				
Positive	*1 (Ref)*			
Negative	2.1 (0.5–9.4)	0.2		
No data entry	1.8 (0.4–7.8)	0.4		
Baseline serum HCV RNA	1 (1–1.3)	0.3		
DAAT regimen				
SOF/VEL	*1 (Ref)*	0.06		
SOF/DCV	0.4 (0.1–1)			
Baseline ALT	1 (0.9–1)	0.9		
Baseline AST	0.9 (0.9–1)	0.6		
Non-invasive Fibrosis Evaluation in Hepatitis C (FIB-4)				
Cirrhosis highly likely	*1 (Ref)*		*1 (Ref)*	
Indeterminate	0.7 (0.4–1.5)	0.5	1.6 (0.6–4.2)	0.3
Cirrhosis less likely	1.5 (0.7–3.1)	0.2	3.7 (1.2–11.5)	**0.02**

HBsAg, Hepatitis B surface antigen; HIV, Human immunodeficiency virus; HCV, Hepatitis C virus; ALT, alanine aminotransferase; ASP, Aspartate Aminotransferase; SOF/DCV, Sofosbuvir/Daclatasvir; SOF/VEL, Sofosbuvir/Velpatasvir.

Significant values are in [bold].

## Discussion

Real-world data for treatment programs in SSA is hard to locate in the published literature. Unlike participants in phase 3 randomized controlled trials (RCTs); real-world study cohorts typically include patients with unfavorable conditions. Furthermore, DAAs outcomes can be compromised by clinicians’ limited expertise^25^. In this study, all patients had unknown genotypes and unknown fibrosis status. Although consistent details were not available for all patients, the overall SVR rate was 116 (92.8%) for a subset of patients. These results are in line with data from multiple seminal RCT studies (the Phase 3 ASTRAL-1, ASTRAL-2, ASTRAL-3 and ASTRAL-5 trials and POLARIS-3 trials); which reported SVR rates of 93–100%^26^. Even more important, other real world studies and RCTs have similarly shown that DAAs are well tolerated by patients^27^.

Where possible, we computed FIB-4 and APRI scores and evaluated SVR for SOF/VEL and SOF/DCV along cirrhosis strata. In this analysis, our results demonstrated that patients with FIB-4 score < 3.25 had higher SVR (94.5%) compared to patients with FIB-4 score > 3.25(90.5%) for both regimens. Generally, our findings are consistent with the observation that fibrosis may not compromise SOF/VEL and SOF/DCV efficacy, but decompensated cirrhosis may undermine SVR^28^. To illustrate these points, Abdul and colleagues noted a significant difference in treatment outcomes between patients with disparate Child-Turcotte-Pugh (CTP) classification (95.5% in CTP A vs. 90.8% in CTP B, *p* value  = 0.010)^29^. Literature also suggests that specific HCV genotypes (e.g., HCV-GT4 subtype 4k, 4q, 4p, and 4r) and the emergence of resistance-associated mutations (RAM)^30^ may contribute to virological failure. Unfortunately, the possible contribution of these factors to SVR rates in this population is difficult. Therefore, further studies will be required to address this gap.

Remarkably, most of the non-responders had high FIB-4 scores, relatively low PLT count (thrombocytopenia can be a surrogate marker of portal hypertension), and high ALT and AST (see “Supplementary data” page 1). Many of these findings align with previous literature which suggested that decompensated liver cirrhosis (CTP B and C), and elevated transaminase levels, among others, are associated with lower SVR^31,32^. Interestingly, post-treatment AST, ALT, FIB-4 score, and APRI score were significantly lower in a sub-set of patients. Furthermore, PLT counts demonstrated limited improvement following treatment. Much of this information concurs partially with literature relating to the clinical benefits of SVR^31–33^ such as the possible restoration of the liver functional reserve^34^. Altogether it’s our conclusion that for most patients, genotype-blind treatment with SOF/VEL or SOF/DCV regimens is largely satisfactory. Importantly, this outcome reinforces the fact that these regimens can have utility in population-level scale-up measures directed at the elimination of HCV in resource-poor settings in SSA.

Despite the favorable SVR rates, mortality rate was high [18 deaths (7.5% (95% CI 1.7–4.1)] with a significant proportion of deaths occurring in the first 8 weeks after initiation of treatment. The high mortality rate observed in this cohort is probably linked to late presentation of patients. Indeed, patients in this cohort were older (Median (IQR): 60 years (50–69 years) suggesting long-term exposure to HCV. At present, reports suggest that HCV-related cirrhosis can be observed in 5–20% of patients after 20–30 years of chronic infection^35^. Others have noted that cirrhotic patients are at high risk of hepatic decompensation (27.7–39.5% risk over five years) and hepatocellular carcinoma (HCC) (2.8–7.4% in the first year, and 8–16.1% over 5 years) and liver-related mortality^36^. In most countries in SSA, liver transplantation and the cost associated with the management of patients with End-Stage Liver Disease (ESLD) is prohibitive, therefore, the condition is invariably fatal. This implies that screening of all at-risk populations is a more affordable option for most countries in the region. Therefore, the need for early detection or scale-ups in screening/case-finding along with robust treatment of patients should be prioritized.

In general, experts agree that determination of the severity of liver fibrosis is a challenging but essential component of HCV management^28^. Highlighting this issue, some have noted that limited provider experience; lack of technology for fibrotic staging (e.g. Fibroscan), confirmatory HCV-RNA testing/or genotype determination, as well as clinical chemistry infrastructure, is compromising treatment in LMIC in SSA^37^. Our data corroborate this position. First, the number of hepatologists or specialized internists is severely limited in Eritrea. Clearly, the lack of clinical expertise may compromise hepatic staging-informed care or management of advanced fibrosis/cirrhosis (an outcome which appears to be common in this setting). In addition, confirmatory HCV-RNA testing is highly centralized and periodic reagent stock-outs have been reported. More importantly, the existing HCV/HBV standard-of-care guidelines for Eritrea highlight the possible use of APRI, FIB-4 score, and liver elastography for fibrosis determination without specifying the preferred approach. On the latter, we can conclude that the FIB-4 score appears to have a good discriminatory capacity for non-SVR and likelihood of mortality. Overall, better diagnostic performance for FIB-4 score has been reported by multiple investigators^37^.

Lastly, it should be noted that successful completion of treatment is critical for long-term HCV elimination goals. Previous work has shown that LTFU-associated DAA treatment interruptions can be linked to avoidable morbidity and mortality, increased health care costs, preventable HCV transmission, and the development of drug resistance mutations. However, despite overwhelming evidence demonstrating the importance of retention in care; our data demonstrate that LTFU was disproportionately high (67(28.1%, 95% CI 22.3–33.9) and that it was the most important non-virological reason for non-attainment of SVR. This outcome stands in stern contrast to reports from a study in Rwanda which reported no LTFUs^38^. Low proportions of LTFUs were also reported by workers in other countries^39,40^. At present, we have to concede that the observed results were largely unexpected because treatment in these facilities is offered *gratis* or with minimal out-of-pocket cost. This aside, it should be noted that a significant number of these patients were categorized as LTFUs. It is likely that many of these patients were not cured and had some level of viremia when they left care. From a public health concern, this outcome can undermine the progress towards the achievement of HCV eradication.

Although consistent details were not available for most patients on a large number of factors with a potential link to LTFU; several variables independently demonstrated significant effects on LTFU rate—treatment center, years of enrollment (2020), and cirrhosis status were independent predictors of LTFU. The link between years of enrollment and LTFU is probably connected to the COVID-19 pandemic and the lockdowns of 2020. On the other hand, the connection between LTFU and treatment centers (Hospital B) may be connected to proximity to laboratories services. For example, viral load testing center is located near Hospital A but is at a considerable distance from the hospital B (see Fig. 1). Moreover, difference in quality of services in either institution may also account for the difference in LTFU. In general, the number of specialized clinicians is severely limited in hospital B. Interestingly, patients who had less likelihood of cirrhosis (FIB-4 score < 1.45) had higher hazards of LTFU. The health belief model predicts this outcome—the perception that a disease is not dangerous can undermine health seeking behavior. Poor patient awareness, cost of treatment/lack of insurance, housing instability/or address changes and distance to care facilities can also drive the incidence of LFTUs^41^. Altogether, our result underscores the fact that even in settings where DAA treatment and essential laboratory services are offered *gratis* or with minimal out-of-pocket fee; LTFU can still be a formidable barrier.

## Limitations of the study

Although this study addresses an important information gap on HCV literature in SSA, it has some limitations. Firstly, poor documentation of baseline history, laboratory data and post-treatment surveillance data undermined our analyses. Lastly, FIB-4 and APRI scores have not been validated for populations in SSA.

## Conclusion

These results provide the first primary data on treatment outcomes in two HCV treatment programs in Eritrea. Important insights included the fact that SOF/VEL and SOF/DCV are highly effective even in settings where genotypes are unknown. Secondly, mortality rates were relatively high, an outcome that was largely associated with the large proportion of patients with cirrhosis. The high incidence of LTFU was surprising given that care was offered for free. Therefore, more work is needed to monitor and understand the factors behind the high proportion of LTFU in this setting. Going forward, more emphasis should be placed on decentralization of care services and better monitoring during and post-EOT.

### Supplementary Information

Supplementary Information.

## Data Availability

The dataset supporting the conclusions of this article is available from the corresponding author on reasonable request.

## Abbreviations

aHR: Adjusted hazards rate
ALT: Alanine aminotransferase
APRI: AST level with platelet ratio index
AST: Alanine aminotransferase
CHC: Chronic hepatitis C
cHR: Crude hazards rate
CI: Confidence interval
DAA: Direct acting antivirals
DCV: Daclatasvir
EOT: End of treatment
FIB-4: Non-invasive fibrosis evaluation in hepatitis C
HBsAg: Hepatitis B surface antigen
HBV: Hepatitis B virus
HCV: Hepatitis C virus
Hgb: Hemoglobin
HIV: Human immunodeficiency virus
IQR: Interquartile range
LMIC: Low and middle income countries
LTFU: Loss to follow up
Plt: Platelets
RCT: Randomized controlled trial
RNA: Ribonucleic acid
SOF: Sofosbuvir
SSA: Sub-Saharan Africa
SVR: Sustained virological response
TB: Tuberculosis
VL: Viral load
WHO: World Health Organization
