# Supplementary material for: Real-world treatment outcome of direct-acting antivirals and patient survival rates in chronic hepatitis C virus infection in Eritrea
Source: Sci Rep. 2023 Nov 27;13:20792. doi: 10.1038/s41598-023-47258-7 (PMC10682448; doi:10.1038/s41598-023-47258-7)
Supplement: Supplementary file 1 — Supplementary Information. [file 41598_2023_47258_MOESM1_ESM.doc]

**Supplementary Table 1:** Characteristics of virologic non-responders

| **Gender** | **Enrollment year** | **Age at enrollment** | **Address** | **DAA** | **AST** | **ALT** | **Platelet count** | **Initial serum HCV/RNA** | **APRI** | **FIB- score** |
| --- | --- | --- | --- | --- | --- | --- | --- | --- | --- | --- |
| **male** | 2020 | 69 | Central zone | SOF/VEL | 54 | 15 | 194 | 11008 | 0.62 | 4.96 |
| **female** | 2020 | 59 | Central zone | SOF/VEL | 23 | 55 | 192 | 1300000 |  | 0.95 |
| **male** | 2021 | 65 | Central zone | SOF/VEL | 263 | 110 | 150 | 298749 | 3.9 | 10.87 |
| **male** | 2020 | 60 | Central zone | SOF/VEL | 52 | 69 | 254 | 687714 | 0.45 | 1.48 |
| **male** | 2021 | 70 | Central zone | SOF/VEL | 47 | 37 | 120 | 30081 | 0.87 | 4.51 |
| **female** | 2019 | 67 | Central zone | SOF/DCV | 44 |  | 197 | 4950000 | 0.5 |  |
| **male** | 2021 | 65 | Central zone | SOF/VEL | 263 | 110 | 150 | 298749 | 3.9 | 10.87 |
| **male** | 2020 | 80 | Outside central zone | SOF/DCV | 28 | 41 | 138 | 3377046 | 0.45 | 2.5 |

Abbreviations: ALT: alanine aminotransferase; AST: Aspartate Aminotransferase; SOF+DCV: Sofosbuvir+Declatasavir; SOF+VEL: Sofosbuvir+Velpatasavir.

**List of tests performed on chronic hepatitis C patients enrolled in the study as per local guideline**

Pre-treatment assessments recommended in the guideline include Complete blood count (CBC), Liver function enzymes (Aspartate aminotransferase (AST), and Alanine aminotransferase (ALT)), kidney function test (creatinine (Cre), total bilirubin (TBIL), coagulant prothrombin (Prothrombin time (PT)), partial thromboplastin time (PTT) and International Normalized Ratio (INR)), serum HCV viral load (COBAS® AmpliPrep/COBAS® TaqMan® HCV RNA assay), HBsAg testing and HIV screening in addition to USG of the liver, adherence – pill count at 4 and 8 weeks, and drug toxicity evaluation. After initiation of DAA, HCV RNA titer is quantified at 12 and 24 weeks.
